# In vitro sensitivity to dasatinib in lymphoblasts from a patient with t(17;19)(q22;p13) gene rearrangement pre-B acute lymphoblastic leukemia

**DOI:** 10.1002/pbc.23383

**Published:** 2012-09

**Authors:** Jason M Glover, Marc Loriaux, Jeffrey W Tyner, Brian J Druker, Bill H Chang

**Affiliations:** 1 Division of Pediatric Hematology and Oncology, Department of Pediatrics, Knight Cancer Institute, Doernbecher's Childrens Hospital, Oregon Health & Science UniversityPortland, Oregon; 2 Division of Hematopathology, Department of Medicine, Knight Cancer Institute, Oregon Health & Science UniversityPortland, Oregon; 3 Division of Hematology and Medical Oncology, Department of Medicine, Knight Cancer Institute, Oregon Health & Science UniversityPortland, Oregon; 4 Howard Hughes Medical Institute, Oregon Health & Science UniversityPortland, Oregon

**Keywords:** ALL, dasatinib, E2A-HLF

## Abstract

Patients with t(17;19) acute lymphoblastic leukemia (ALL) have a dismal prognosis even with the most intensive current therapies that include stem cell transplant. We present the case of a patient with t(17;19)(q22;p13) gene rearranged B-cell precursor ALL whose lymphoblasts were found to have significant in vitro sensitivity to dasatinib. The patient tolerated the addition of dasatinib with combination therapy and remained in remission for over nine months until his recurrence. Therefore, future studies will be needed to interrogate whether dasatinib has any therapeutic benefit in children with t(17;19) B-cell precursor ALL.

## INTRODUCTION

Significant advances in outcomes for childhood leukemia have been made over the past half-century with cure rates approaching ninety percent 1. Nonetheless, certain genetic subtypes of acute lymphoblastic leukemia (ALL) continue to have poor outcomes. Among the most unfavorable subtypes is B-cell precursor ALL containing a 17;19 translocation, as there are no published reports of children surviving this specific malignancy. This disease subtype is extremely rare, accounting for ≤1% of all childhood B-cell precursor ALL 2. The t(17;19)(q22;p13) gene rearrangement yields a fusion product *E2A-HLF* which encodes a chimeric transcription factor that consists of the transactivation domain of *E2A* linked to the bZIP DNA-binding and protein dimerization domain of hepatic leukemia factor, *HLF* 3, 4. Type I rearrangement arises from a breakpoint within intron 13 of *E2A* and intron 3 of *HLF* and is associated with disseminated intravascular coagulation (DIC). Type II rearrangement has a breakpoint located at intron 12 of *E2A* and intron 3 of *HLF* and is associated with hypercalcemia from elevation of a parathyroid hormone related peptide 5, 6.

Because of its rarity and poor prognosis, there are no specific recommended therapies for patients with this disease. Patients who achieve remission with no evidence of disease continue to recur and die of disease even with stem cell transplant as consolidation therapy 7. Previous data would suggest this chemotherapy resistance may be due to enhanced expression of the drug efflux gene *ABCB1* 8. Therefore, novel therapies are clearly needed to improve outcomes.

We recently developed an in vitro assay using a panel of small molecule inhibitors to identify patient specific targeted drugs. We employed this assay on the diagnostic marrow sample of a patient who presented to our institution with t(17;19) ALL. Interestingly, his leukemic blasts were very sensitive to several small molecule inhibitors highlighting pathways such as PI3K/AKT. Many of these compounds are tool compounds to interrogate selective pathways and are not in therapeutic trials. Meanwhile, one interesting observation was the sensitivity to the FDA approved drug, dasatinib suggesting the possibility of the addition of this drug to the patient's treatment regimen.

## CASE REPORT

**Figure 1:**
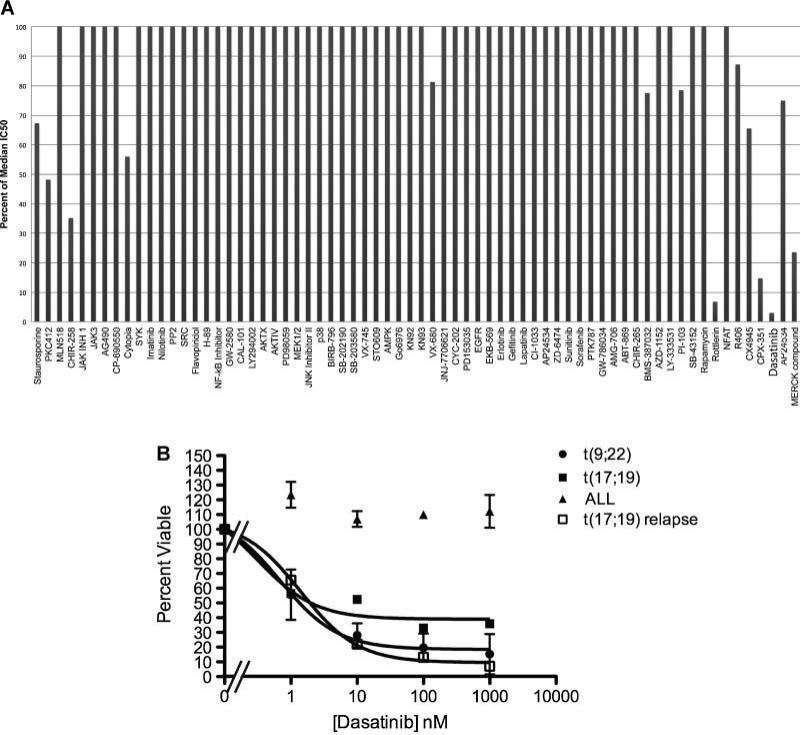
Sensitivity to small molecule inhibitors. A: Inhibitor assay. Currently, we have Institutional Review Board approval for obtaining extra samples for interrogation by our inhibitor assay. Informed consent was obtained for collection of extra bone marrow sample for drug testing. Leukemic cells were isolated using Ficoll-Paque, and run on our laboratory's small molecule inhibitor panel. 5 × 10^4^ cells were plated into each well on a 96 well plate containing multiple small molecule inhibitors in 4 logarithmic concentrations. The cells were cultured with the drug for three days, at which time cell viability was determined using a colorimetric assay (Cell Titer Aqueous One Solution Cell Proliferation Assay; MTS; Promega, Madison, WI). All values on each plate were normalized to the mean of seven wells containing no drug. The IC_50_ of each drug for this patient was calculated compared to the no drug wells and compared with median IC_50_ values generated by profiling primary cells obtained from over 150 different leukemia patients. The normalized IC_50_ values for this individual patient were expressed as percent of median IC_50_. Each bar represents the IC_50_ of a different small-molecule kinase inhibitor for this individual patient sample as a percent of the median IC_50_ of 150 patients, such that low values indicate this individual patient sample was significantly hypersensitive to a given drug. B: Dose–response curve for dasatinib. Representation of the dose-response curves used to generate the IC_50_ for the patient samples. Circle, patient with Ph + ALL; closed square, the patient with t(17;19); open square, patient with t(17;19) at relapse; triangle, patient with non-t(17;19)/non-Ph + ALL—unresponsive to dasatinib.

Our patient presented at the age of ten years with symptoms of vomiting, pallor, fatigue, pancytopenia, hypercalcemia (total calcium level of 15.5 mg/dL) and renal insufficiency. His initial white blood cell count was 10,900 per cubic mm with circulating lymphoblasts. The diagnostic bone marrow aspiration showed 95% precursor B-cell lymphoblasts with a more mature immunophenotype: CD10, CD19, CD22, cCD79a, HLA-DR, and TdT. Cytogenetic evaluation identified the following complex karyotype: 46,XY,t(17;19)(q22;p13.3)[2]/47∼50,idem,+22[cp3]/46,idem,del(6)(q1?1.2q2?2),add(9)(p?13)[5]/44∼45,idem,add(X)(p22.1),add(1)(p3?4),-9,add(12)(q2?2)[cp2]/46,XY[17]. He underwent standard 4-drug induction chemotherapy with vincristine, doxorubicin, asparaginase, and prednisone. At presentation, extra bone marrow aspirate was obtained with informed consent and examined by our inhibitor assay (Fig. 1A). This assay determined that the patient's lymphoblasts were sensitive to multiple small molecule inhibitors. Interestingly, one of the compounds to which the cells were hypersensitive was the FDA approved drug dasatinib (Sprycel, Bristol Meyer Squibb). The induction course was unremarkable with a resolution of his hypercalcemia in less than 7 days. He also had no documented coagulopathy. At the end of induction he was found to have 6% blasts in marrow aspirate by morphology and 13% blasts by flow cytometry. He proceeded with intensive chemotherapy previously developed by The Children's Oncology Group (COG) for very high risk ALL (AALL0031) 9. One month into therapy there was persistent cytogenetic evidence of disease at 6% by fluorescent in situ hybridization (FISH). Following parental approval, dasatinib was added to his chemotherapy regimen at 60 mg/m^2^ daily. One month into this combination therapy he was found to be in complete remission with no evidence of disease by FISH, flow cytometry or morphology. He tolerated the chemotherapy regimen and dasatinib for nine months well with excellent quality of life. He showed no evidence of toxicities with the addition of dasatinib and was able to continue with therapy without any extended delays. Nine months into therapy while continuing chemotherapy and dasatinib, he acutely developed hypercalcemia, abdominal pain, and circulating lymphoblasts. Cytogenetic evaluation revealed a similar complex karyotyope with t(17;19); 45,Y,add(X)(p22.1),add(1)(p3?4),del(6)(q1?1.2q2?2),-9,add(12)(q2?2),t(17;19)(q22;p13.3)[1]/46,XY[24]. His marrow lymphoblasts at relapse remained sensitive to dasatinib in our small molecule assay (Fig. 1B). He was taken off all medications in order to be eligible for a clinical trial. One week off dasatinib his disease burden worsened with increasing serum calcium and worsening renal insufficiency. Therefore, re-induction was attempted with clofarabine, cyclophosphamide, and etoposide. He developed significant capillary leak syndrome, hypercalcemic renal failure, prolonged neutropenia, and systemic *Candida parapsilosis*. He died of multiple organ system failure 11 months after his initial diagnosis.

## DISCUSSION

We report a novel finding on a patient with t(17;19) whose lymphoblasts showed in vitro sensitivity to dasatinib at low nM range. Dasatinib's cytotoxic mechanism in this case is unclear at present. Cytotoxicity may be due to the activity of dasatinib on SRC family kinases rather than on ABL as in cases of t(9;22) ALL. Signaling through the SRC family kinases HCK, LYN, and FGR has also been shown to be required for leukemic transformation in t(9;22) ALL 10. Perhaps there are similar SRC kinase requirements for oncogenic survival in leukemic cells with a t(17:19) translocation.

The patient sample's immunophenotype would suggest a more mature lymphoblast population similar to t(1;19) samples 11 that is known to express the pre B-cell receptor. Prior studies have shown the importance of B-cell receptor signaling in the more mature, chronic lymphocytic leukemia 12. B-cell receptor signaling is dependent on the SRC kinase family 13. This dependence on the B-cell receptor can be targeted through treatment with dasatinib through inhibition of Lyn and BTK. An intriguing concept would be that our patient's sample is dependent on the pre B-cell receptor and that dasatinib may be affecting lyn activity. This would be in agreement with the sensitivity seen with rottlerin from our sample (Fig. 1A) described by Guo et al. of B-cell receptor signaling 13. The IC_50_ for dasatinib for our patient's sample was similar to that of blasts from a patient with t(9;22) ALL (Fig. 1B) suggesting that dosing of dasatinib used to treat t(9;22) ALL may reach therapeutic efficacy in our patient with t(17;19). Further studies will be needed to understand the mechanism of sensitivity to dasatinib in our patient lymphoblasts and whether these lymphoblasts are a model for other t(17;19) containing lymphoblasts.

The COG AALL0031 trial that combined intensive chemotherapy with the small molecule inhibitor imatinib mesylate has shown a dramatic increase in early survival for patients with t(9;22) ALL (3 year EFS of 88%) 9. The latter study demonstrated clinical tolerability and efficacy of adding small molecule inhibitors to intensive chemotherapy. Dasatinib is a multi-targeted tyrosine kinase inhibitor that is more potent than imatinib against analogous targets, such as ABL as well as a SRC inhibitor. This drug is currently FDA approved for the treatment of CML and t(9;22) ALL. It has been proven to be tolerable and efficacious in adults with t(9;22) ALL in combination with chemotherapy 14, and is currently being studied in children with t(9;22) on the COG (AALL0622) trial. Our results may help develop the foundation for the addition of patients with t(17;19) ALL to be enrolled on AALL0622 or similar trials.

Our patient suffered a recurrence in the presence of dasatinib despite persistent in vitro sensitivity. It is unclear as to the mechanism of dasatinib resistance in our patient. Clearly the blast population in vitro remained sensitive to dasatinib. It was evident that the patient was receiving his medications without compliance issues. One possibility for this phenomenon would be the host pharmacokinetics. We do not have any direct evidence that the dose of dasatinib was achieving inhibitory effects at the time of relapse and was not able to attempt to increase the dose at the time of recurrence. Another possibility is that studies have shown that cell lines with t(17;19) over express *ABCB1* 8 allowing for drug resistance through drug-efflux. Clearly standardizing the therapy for patients with this rare disease will be needed to advance their outcomes.

## AUTHORSHIP CONTRIBUTIONS

Contribution: J.M.G. and J.W.T. performed the assays. J.W.T., M.L., and B.J.D. designed the assays. J.M.G. B.H.C, J.W.T, M.L., and B.J.D. wrote the manuscript.
